# Incipient Decay

**Published:** 1899-01

**Authors:** 


ARTICLE V.

### INCIPIENT DECAY.

Both among patients and dentists, it is sometimes a question whether incipient decay should be interferred with. Both are apt to look on the most extensive decay as that which should receive first and most thorough attention.

If there must be a discrimination; I would advise, by all means, that the worst teeth be left to rot, while those which can be most cheaply and easily and surely saved should receive our first and best attention.

"Doctor, please look at my teeth, and tell me what work is really the most important. I can't endure or afford to have much work done; but I suppose there is some that must be attended to."

What dentist has not been accosted somewhat in this manner? And how many dentists forthwith, "and, of course," begin to hunt for the teeth nearest destruction?

We admit it would be hardly right to neglect the worst decayed teeth because of the extra time and pain and expense attending their restoration; but really, if any must be neglected, would it not be better to let these go to the spitton while we give attention to those more easily restored to a healthy condition ?

We know, too, that the neglect of the worst jeopardizes the least affected. But we are not advocating the neglect of any. We believe the dentist should be thorough with all, and condemn none that by any fair means can be restored to health. But we do say it is of the utmost importance for the safety of all, to see that incipient decay be as thoroughly and promptly treated as the most advanced caries.

And this should be impressed on the attention of the patient. It should take but little time or argument to show them that decay in one tooth must injure others, and that incipient or superficial decay is much easier removed than if allowed to become deeper-seated. I have often prevented much decay, suffering and expense by removing slight attacks of decay, and then thoroughly polishing the affected surface, especially approximal surfaces and softened spots near the gums. A judge came to me once with decay between every one of his upper front teeth. "Ah," said I, "you are just in time. I can remove every decay without leaving a single cavity to be filled." His teeth were quite crowded. I separated them with rubber and then filed the approximal surfaces until the decay was entirely obliterated. I then polished and hardened them, and giving him an old worn-out separating file told him to once in a while, by and by, to pass it over these surfaces. He had also softened surfaces along the margin of the gums, which I treated in the same way. Many years afterward he came back with these teeth still free from caries, saying, "I have all these years been my own dentist by the use of that old file." Perhaps I ought to have told this in a whisper; for our modern scientific brethren will very much disapprove of such a course. And I must myself admit it would not do for common practice. But I do believe many a softened surface next to the gums, and superficial cavity, might be treated without the necessity of a filling. It may well be called preventive practice; and prevention is better than cure.

I was once working on the teeth of the wife of a noted lawyer, when I discovered what seemed to be but a slight decay on the posterior approximal surface of the lower first bicuspid.

"Well," said she, "I shall be here again in six months. I'll risk it till then."

"I think that will be all right," I replied; "and so of these three or four other superficial decays, I have attended to the most important."

Eight or nine months after this, she came in to say there was an uneasy sensation in this bicuspid. A month later I looked over her teeth again, the bicuspid among the others.

"Nothing serious," said I, and after attending to three or four other small decays, came to my old friend the bicuspid. It was so crowded, I worked a piece of rubber between and made another appointment. To my astonishment, I found the pulp exposed, and that in the second bicuspid nearly so—all this without more than the casual appearance of superficial decay. That taught me a lesson for the future. It was three months before the tooth was dismissed, and even then to give occasional trouble.—EDITORIAL in *Dental Brief*.

